# Contrasting distribution patterns between aquatic and terrestrial *Phytophthora* species along a climatic gradient are linked to functional traits

**DOI:** 10.1038/s41396-018-0229-3

**Published:** 2018-08-02

**Authors:** Miguel A. Redondo, Johanna Boberg, Jan Stenlid, Jonàs Oliva

**Affiliations:** 1 Department of Forest Mycology and Plant Pathology, Swedish University of Agricultural Sciences, Box 7026, 750 07 Uppsala, Sweden; 2 Department of Crop and Forest Sciences, Agrotecnio center CERCA, University of Lleida, Alcalde Rovira Roure, 191, 25198 Lleida, Spain

## Abstract

Diversity of microbial organisms is linked to global climatic gradients. The genus *Phytophthora* includes both aquatic and terrestrial plant pathogenic species that display a large variation of functional traits. The extent to which the physical environment (water or soil) modulates the interaction of microorganisms with climate is unknown. Here, we explored the main environmental drivers of diversity and functional trait composition of *Phytophthora* communities. Communities were obtained by a novel metabarcoding setup based on PacBio sequencing of river filtrates in 96 river sites along a geographical gradient. Species were classified as terrestrial or aquatic based on their phylogenetic clade. Overall, terrestrial and aquatic species showed contrasting patterns of diversity. For terrestrial species, precipitation was a stronger driver than temperature, and diversity and functional diversity decreased with decreasing temperature and precipitation. In cold and dry areas, the dominant species formed resistant structures and had a low optimum temperature. By contrast, for aquatic species, temperature and water chemistry were the strongest drivers, and diversity increased with decreasing temperature and precipitation. Within the same area, environmental filtering affected terrestrial species more strongly than aquatic species (20% versus 3% of the studied communities, respectively). Our results highlight the importance of functional traits and the physical environment in which microorganisms develop their life cycle when predicting their distribution under changing climatic conditions. Temperature and rainfall may be buffered differently by water and soil, and thus pose contrasting constrains to microbial assemblies.

## Introduction

Plant pathogenic microorganisms pose an increasing threat to natural ecosystems globally [1, 2]. Among plant pathogens, fungal-like species belonging to the genus *Phytophthora* are the causal agents of major disease outbreaks worldwide [3–5]. Several studies have shown that numerous *Phytophthora* species are distributed with plant nursery stock [6, 7]. Nevertheless, once introduced into new ecosystems, not all species manage to establish and spread further. Because of the influence of both biotic and abiotic factors, only ecologically compatible species can establish, reproduce and spread further away from their introduction spot, and form an invasive population [8, 9]. A better understanding of the mechanisms underlying the success or failure of the establishment of *Phytophthora* plant pathogens is needed in order to predict future outbreaks. Although climatic factors have been shown to affect the establishment and distribution of some *Phytophthora* species [10], the extent to which climate is associated with the distribution of plant pathogenic *Phytophthora* species at the genus level is still unknown.

Deciphering the factors driving the global patterns of diversity of living organisms is still an open question in ecology [11]. Climatic factors have been suggested to be important predictors of the global and regional diversity of plants and soil microorganisms, including soil fungal pathogens [12–15]. Changes in diversity are associated with changes in functional diversity because species richness and functional richness are positively correlated. However, as a consequence of different ecological processes, such as environmental filtering, the values of functional diversity can be lower than expected from the decrease of the number of species [16]. Environmental filtering is an ecological process in which environmental factors select for certain functional traits, and consequently, against certain species. Therefore, communities shaped by environmental filtering have a lower functional diversity than expected from the regional pool of species and traits because some trait combinations are not compatible with the environmental conditions [16, 17]. Environmental filtering theory predicts that the environmental filtering would be stronger under harsh climatic conditions than under more benevolent conditions [18], although observations remain inconsistent across studies [16, 19, 20].

The effect of climate on the assembly of communities depends on the responses of each species to changes in climate, which may be ultimately linked to certain functional traits [21–23]. Species of the genus *Phytophthora* show different physiological traits that could play a role in their fitness in different environments. For instance, some *Phytophthora* species can create sexual and/or asexual survival structures, such as oospores, chlamydospores, hyphal swellings, or hyphal aggregations, which allow them to survive under harsh environmental conditions [24, 25]. *Phytophthora* x *alni*, for example, does not produce asexual survival structures and, owing to its hybrid nature, produces high rates of non-viable oospores. The limited production of any survival structures might explain why *P.* x *alni* does not survive after long periods of frost [26] and why it is seldom found in areas with long periods of consecutive days below −5°C [10]. By studying the distribution of functional traits, the community composition of plants along environmental gradients can be predicted [27–29]. Whether distribution predictions based on traits is also possible for plant pathogenic microorganisms is still unknown.

Most *Phytophthora* species complete their life cycle in terrestrial ecosystems, as foliar, root, or stem pathogens (hereinafter referred to as terrestrial *Phytophthora* species) [30]. However, the genus *Phytophthora* also contains several species, mostly belonging to phylogenetic clade 6 or 9, that are considered to be resident in water bodies (hereinafter referred to as aquatic *Phytophthora* species) [30–34]. Aquatic species are seldom isolated from terrestrial environments [30], while terrestrial *Phytophthora* species are commonly found in water bodies, possibly washed out from the catchment areas [35, 36]. Terrestrial and aquatic macroorganisms that inhabit the same geographical region seem to have different drivers of diversity, as shown for trees [37], birds [38], or fishes [39]. In the case of terrestrial and aquatic *Phytophthora* species, water or soil may modulate climatic factors such as temperature or precipitation in different ways. Given the large number of *Phytophthora* species and trait variation within terrestrial and aquatic *Phytophthora* species, they represent a good model system to study whether the patterns of diversity and community assembly across a climatic gradient depend on the physical environment (water or soil) where plant-associated microbes develop their life cycle.

The aim of this work was to study the patterns of diversity and community assembly of *Phytophthora* microorganisms. We hypothesized that: (i) climatic conditions are associated with the diversity and functional diversity of assemblies of the genus *Phytophthora*; (ii) the climatic factors associated with the diversity, trait assembly of *Phytophthora* communities, and environmental filtering, are different depending on whether *Phytophthora* species are preferentially terrestrial or aquatic. To test these hypotheses, we sampled 96 sites distributed across 16 rivers along a gradient of temperature and precipitation in Sweden over two consecutive years. Water bodies constitute a sink of *Phytophthora* propagules washed out from the catchment area. For this reason, surveys monitoring *Phytophthora* plant pathogens have increasingly focused on forest streams [9, 35, 40]. We obtained the *Phytophthora* communities using a novel high-throughput sequencing methodology based on PacBio sequencing of river filtrates. Unlike previously developed methods that have only targeted the internal transcribed spacer 1 (ITS1) region [35, 41], we sequenced both ITS1 and ITS2 regions, obtaining a higher resolution for species identification. We explored the main drivers of the diversity and trait composition of *Phytophthora* communities by studying the association between environmental factors, such as precipitation, temperature, and water chemistry, and the diversity and trait dominance of terrestrial and aquatic communities. To confirm that the effect of climatic factors on trait assembly did not only operate at the community level by affecting only the most dominant species but the majority of species in the community, the analysis was also performed at the species level. We also investigated whether we could observe differences in environmental filtering between terrestrial and aquatic communities.

## Materials and methods

### Sampling procedures

To obtain both the aquatic and terrestrial *Phytophthora* communities, we sampled at 96 sites located in 16 rivers in southern Sweden (for detailed information about the rivers, see [10]). All 96 sites were sampled between August and October in 2013, and again in 2014. We categorized the land use of each sampling site as “urban”, “agricultural”, or “forest”, according to the dominant land use type surrounding the site 500 m up- and downstream. At each site, 12 l of water were collected with a bucket, and filtered through an 8-μm membrane (Merck Millipore, Burlington, MA, USA) attached with a polysulfone filter holder (Schleicher & Schuell, Germany) to the pump of an agricultural hand sprayer. Membranes were replaced every time they became obstructed until all water was filtered. The membranes were stored inside 60-mm Ø Petri dishes at 5 °C for up to 48 h before transportation to the laboratory, where they were then stored at −20 °C until they were processed. To avoid cross-contamination between sampling sites, the pumps were rinsed with 5% sodium hypochlorite and distilled water after sampling at each site. During the survey in 2014, water samples were collected at each sampling site and analyzed within 24 h by the geochemical laboratory of the Department of Aquatic Sciences and Assessment of the Swedish University of Agricultural Sciences (https://www.slu.se/en/departments/aquatic-sciences-assessment) to determine their pH, conductivity, total organic carbon, and total nitrogen concentration.

### DNA preparation for PacBio amplicon sequencing

All samples were prepared for Pacific Biosciences SMRT PSII amplicon sequencing of the complete ITS region. For DNA extraction, filters were cut in half (corresponding to 6 l) and DNA was extracted using the NucleoSpin® Soil kit (Macherey-Nagel, Hoerdt, France). After extraction, DNA was quantified using the NanoDrop spectrophotometer (Thermo Fisher Scientific, Waltham, MA, USA). We prepared aliquots of 0.5, 1, 2, and 5 ng μl^−1^ of each sample to select the optimal sample concentration for PCR. The optimal concentration corresponds to the lowest concentration at which a PCR product was visible in an agarose gel so the saturation phase of the PCR reaction was not reached [42]. The PCR was run using a combination of three primers based on the ITS-based *Phytophthora*-specific primers developed by Drenth et al. [43]. Drenth et al. [43] primers were modified in three sites to correct species-specific mismatches and avoid biases against certain species of *Phytophthora* (Supporting Information Table S1). In order to sequence several samples simultaneously, primers were tagged using 8-base pair long tags constructed using the Barcrawl software [44]. Once the optimal concentration was determined, three replicates of each sample at its optimal concentration were amplified using the following cycling conditions: an initial denaturation step at 94 °C for 5 min, followed by 32 cycles of 94 °C for 30 s, 60 °C for 30 s, and 72 °C for 1 min, and a final elongation at 72 °C for 6 min. The PCR products were pooled and purified using an AMPure kit (Beckman Coulter Inc., Brea, CA, USA). For the library preparation, the DNA of the pool was quantified using a Qubit fluorometer, and equal amounts from each sample were pooled for sequencing. Adaptor ligation and sequencing was performed at SciLifeLab (NGI, Uppsala), where a total of 192 samples were sequenced on 16 SMRT cells.

### Analysis of sequences and taxonomical identification

Sequences were de-multiplexed, filtered, and clustered using the SCATA (http://scata.mykopat.slu.se) pipeline. Quality filtering was performed to remove reads with an average score of <20 or with a base quality of <2. Sequences were screened for primers (using 0.9 as a primer match). The reads were then clustered in operational taxonomic units (OTU) using 0.5% as a maximum distance with the neighbor cluster, and using a penalty of one for mismatch, gap opening, and gap extension. Samples with different tags in the forward and reverse primers were deleted. OTUs were identified by blasting the consensus sequence of each cluster on *Phytophthora*-ID (http://Phytophthora-id.org), and GenBank (http://www.ncbi.nlm.nih.gov/genbank/), establishing a minimum threshold of 98% identity and 85% coverage with curated sequences. When OTUs could not be identified at the species level (<98% similarity with any reference sequences), they were assigned to one of the 10 clades present in the *Phytophthora* genus [45]. For this, the consensus sequence of the OTU was aligned with the ITS reference sequences from *Phytophthora*-ID using a penalty of 400 for gap opening and 50 for gap length. A maximum-likelihood tree was built using 500 bootstrap replications and including all the positions of the sequences; the phylogenetic tree was rooted using two *Phytopythium* spp. (Supporting Information Fig. S1). Mega software (version 7) was used for both the alignment and to construct the tree. Although *Phytophthora* clades are defined using several genes, most of the clades can also be differentiated based on the ITS region [35]. However, OTUs placed in clades that could not be identified based on the ITS region alone were assigned to the clade of the phylogenetically closest reference sequence based on ITS similarity. Reads were considered to belong to the genus *Phytophthora* when they either (i) clustered to an OTU that could be identified at species level, (ii) to an OTU identified at clade level based on the rooted phylogenetic tree, or (iii) to an OTU placed within clades of the *Phytophthora* genus in the rooted phylogenetic tree.

The validity of the primers was tested on a mock community (Supporting Information Methods S1 and Table S2), and by comparing the OTUs list found by sequencing with the list of *Phytophthora* species isolated by baiting for 3 days in the same site of the river from where water was collected in 2014 (Supporting Information Table S3; detailed isolation procedures are described in Redondo et al. [9]).

### Analysis of diversity

**Fig. 1:**
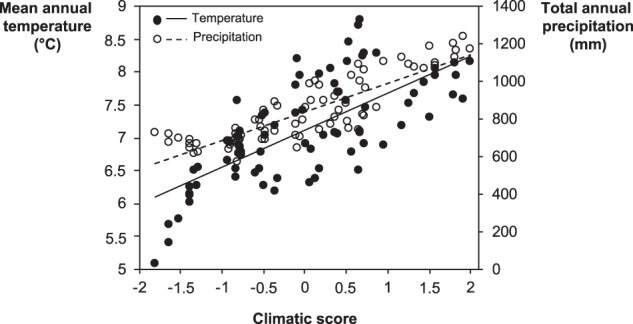
Association between climatic score, mean annual temperature, and total annual precipitation. The mean annual temperature and the total annual precipitation correspond to the average of the last 20 years. Climatic score corresponds to the score of each sampling site on the first axis of a principal component analysis, which included the average values of both mean annual temperature and total annual precipitation of the last 20 years

We analyzed the relationship between the diversity of *Phytophthora* communities and temperature, precipitation, the land use of the sampling site (“urban”, “agricultural”, or “forest”), and water chemistry parameters. All communities were down-sampled to the same number of reads (350 reads per sample) using the rrarefy() function in the Vegan package for R, and we divided the species into two groups: aquatic species and terrestrial species. Aquatic species were those belonging to the phylogenetic clade 6 (since no species from clade 9 were found), which are considered resident in water bodies [30–32]; the rest of the species were considered terrestrial. We calculated species richness, Shannon index, Simpson index, and Pielou’s evenness separately for communities of aquatic and terrestrial species with more than one species using the Vegan package in R. The mean monthly temperature of the air and the mean monthly precipitation for the past 20 years for each sampling site was obtained from the Swedish Meteorological and Hydrological Institute website (luftweb.smhi.se). Temperature and precipitation were positively correlated, therefore we merged all values into a single composite variable called “climatic score” by performing a principal component analysis (PCA) in Canoco 5 (Microcomputer Power, Ithaca, NY, USA) (Fig. 1), as reported in other studies for chemistry data [46]. The ordination score on the first axis of the PCA (gathering 58.9% of the variation) was used as a climatic score for each sampling site. To test how much of the diversity variation was explained by temperature and precipitation separately, we built a generalized or general linear model in R for each diversity parameter, using the mean annual temperature and the total annual precipitation as independent variables, and the diversity parameter as the response. In total for the 2 years, we determined the diversity of 110 terrestrial communities (with 27 and nine OTUs identified at species and clade level, respectively) and 156 aquatic communities (with nine and five OTUs identified at species and clade level, respectively).

### Trait selection and analysis of functional diversity

We studied the association between four functional diversity parameters, namely functional richness (FRic), functional dispersion (FDis), functional evenness (FEve), and the community-level weighted mean (CWM) and five explanatory variables, namely climatic score, precipitation, temperature, the land use of the site, and water chemistry (pH, conductivity, total organic carbon, and total nitrogen) using generalized linear models. Functional parameters were calculated using the FD package in R [47]. For FRIc, FDis, and FEve, only communities with more than three species were included in the analysis, whereas for the CWM, all communities were considered. Functional trait information was compiled for each of the 36 *Phytophthora* species identified at the species level (Supporting Information Table S4). Functional traits were selected based on their link to dispersal mode (persistent/caducous sporangia), taxonomic classification (clade), production of sexual (mode of reproduction) or asexual survival structures, cardinal temperatures for growth (minimum, optimum, and maximum), and ability to colonize the host (host and plant tissue range), as described in Redondo et al. [9]. To address any inconsistencies in the literature regarding functional trait information, we followed the criteria for all species as explained in Supporting Information Methods S2. In total, for both years, we studied the functional diversity of 56 terrestrial communities (with 27 OTUs identified at species level) and 145 water communities (with nine OTUs identified at species level), and the CWM of 164 terrestrial and 192 water communities.

We tested the association between functional traits and climate at both the community and species level. At the community level, we analyzed the relationship between the dominant trait of the community (obtained from the CWM) and the climatic score using linear models, using the trait as an independent variable and the climatic score as the response. At the species level, we tested the association between the functional trait of each species and the species climatic score. The species climatic score was calculated as the climatic score of the locations where the species with more than one occurrence over the 2 years of the survey were present, weighted by the proportion of sequences of the species at the sampling site.

We quantified the amount of variation of the distribution of each functional trait explained by temperature and precipitation separately by performing a generalized linear regression. In these models, we used the proportion of the community for the different levels of each functional trait as a response variable, and the mean annual temperature and total annual precipitation as explanatory variables. All analyses mentioned above were performed in R.

### Environmental filtering analysis

We searched for a signal of environmental filtering by testing whether the functional richness of the community was lower than the null expectation resulting from a decrease of species richness, following the same procedure as Lamanna et al. [16]. For this, we simulated 5000 communities for each number of species richness observed in our study (communities with between three and 12 *Phytophthora* species) by subsampling without replacement from the pool of species (a total of 27 terrestrial species, and nine aquatic species). A value of functional richness was considered significantly lower than the null expectation when it was below the lower 90% quantile of the simulated communities (one-sided *t*-test at *P* *<* 0.05). Only functional richness was used in this analysis because this is the only diversity parameter that does not depend on the relative abundance of each species, so it allowed the construction of simulated communities by randomly subsampling species without considering their abundances.

## Results

### Sequencing output

After quality filtering, we obtained 228 913 PacBio reads, of which 169 821 (74.2%) were *Phytophthora* reads. A total of 89 457 (52.7%) *Phytophthora* reads corresponded to data collected in 2013, and 80 364 (47.3%) from the data collected in 2014. The average number of *Phytophthora* reads/site and year was 884. The *Phytophthora* reads clustered into 64 OTUs, from which 36 OTUs were identified at the species level (81.5% of total *Phytophthora* reads) (Table 1), 14 OTUs were identified at the clade level (4% of total *Phytophthora* reads), and 14 OTUs could not be assigned to any specific clade (14.5% of total *Phytophthora* reads) (Supporting Information Fig. S1). The OTUs identified at species level belonged to the clades 1, 2, 3, 4, 6, 7, 8, and 10. Within OTUs identified at the species or clade level, aquatic communities (i.e., OTUs belonging to clade 6) represented 83.8% of the total *Phytophthora* reads, and terrestrial communities (i.e., OTUs belonging to clades other than clade 6) represented the 16.2%. The most abundant aquatic species were *Phytophthora lacustris*, *Phytophthora gonapodyides*, and *Phytophthora bilorbang* (corresponding to the 57.4%, 27.7%, and 12% of total reads of aquatic species, respectively). The most abundant species within terrestrial communities were *Phytophthora gallica*, *P. alni* (species complex), and *Phytophthora cactorum* (corresponding to the 53.6%, 16%, and 10.4% of total reads of terrestrial species, respectively) (Table 1).

**Table 1:** OTUs identified at species level with its corresponding clade, proportion of reads, and occurrence

*Phytophthora* species	Clade	% of total reads^a^	Number of sites present^b^
**Aquatic** ***Phytophthora*** **spp. (Clade 6 spp.)**			
*P. bilorbang*	6	12	88
*P. chlamydospora*	6	0.01	5
*P. gonapodyides*	6	24.4	92
*P. gregata*	6	0.7	11
*P. lacustris*	6	50.4	95
*P. megasperma*	6	0.07	6
*P. pinifolia*	6	0.06	4
*P. riparia*	6	0.2	19
*P. rosacearum*	6	0.1	5
**Terrestrial** ***Phytophthora*** **spp. (Non-clade 6 spp.)**			
*P. alni*	7	1.9	44
*P. brassicae*	8	0.02	5
*P. cactorum*	1	1.3	29
*P. cambivora*	7	0.04	9
*P. capensis*	2	0.007	1
*P. cichorii*	8	0.006	2
*P. cryptogea*	8	0.07	4
*P. drechsleri*	8	0.02	5
*P. europaea*	7	0.5	18
*P. fragariae*	7	0.3	21
*P. fragariaefolia*	7	0.01	4
*P. gallica*	10	6.5	73
*P. iranica*	1	0.03	8
*P. niederhauserii*	7	0.004	1
*P. pisi*	7	0.06	7
*P. plurivora*	2	0.01	5
*P. porri*	8	0.009	3
*P. primulae*	8	0.002	1
*P. pseudosyringae*	3	0.5	32
*P. psychrophila*	3	0.006	1
*P. quercina*	4	0.04	1
*P. ramorum*	8	0.01	1
*P. sansomeana*	8	0.1	9
*P. sojae*	7	0.02	2
*P. syringae*	8	0.4	32
*P. trifolii*	8	0.1	9
*P. uliginosa*	7	0.02	2

^a^The total number of reads correspond to those belonging to OTUs that were identified at species level

^b^Number of sites where a species was found over the 2 years of the survey. Number of total sites = 96

### Diversity and functional diversity of terrestrial *Phytophthora* species

**Fig. 2:**
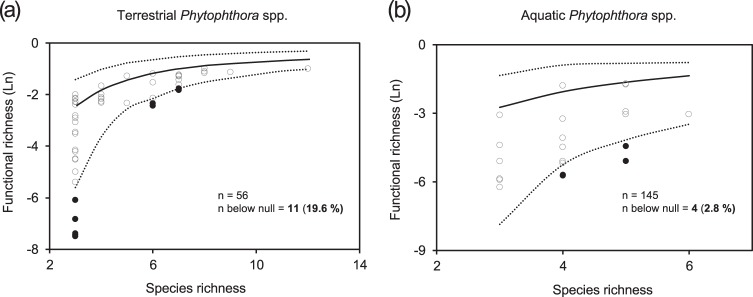
Association between species richness and functional richness for simulated **a** terrestrial and **b** aquatic *Phytophthora* communities. The lines correspond to the average (solid black line) and the 90% quantile (dotted lines) of the 5000 simulations of functional richness for each number of species richness. Solid black circles correspond to communities with significantly lower functional richness than the null-model simulated communities (one-tailed *t*-test, *P* < 0.05). Some communities had the same value of functional richness, and therefore some solid dots are overlapped

For terrestrial *Phytophthora* communities, we found an association between environmental factors and diversity and functional diversity. Using the climatic score (temperature and precipitation combined), we found a decrease of diversity and functional diversity among terrestrial communities with decreasing temperature and precipitation (Supporting Information Table S5). When both factors were included as separate explanatory variables for diversity and functional diversity parameters, precipitation accounted for more variation than temperature (Table 2). The species richness, Shannon index, Simpson index, functional richness, and functional evenness of the terrestrial communities decreased with decreasing precipitation (Table 2). The functional richness was lower than the null expectation, pointing to environmental filtering, at 19.6% of the sites (11 out of 56) (Fig. 2a). The majority (64%) of communities with signs of environmental filtering were composed of only three species. Neither the land use of the sampling site, nor any of the water chemistry parameters were associated with the diversity or functional diversity of terrestrial communities (Table 2 and Supporting Information Table S6). The number of species and values of alpha diversity were higher among the samples collected in 2014 than in 2013 (Table 2).

**Table 2:** Variation of diversity and functional diversity of terrestrial and aquatic *Phytophthora* communities explained by temperature, precipitation, the land use of the site (urban, agricultural or forest), and the year of the survey

	Species richness		Shannon index		Simpson index		Pielou’s evenness		Functional richness		Functional dispersion		Functional evenness	
	Chi-square	*P*	*F*- value	*P*	Chi-square	*P*	Chi-square	*P*	Chi-square	*P*	Chi-square	*P*	Chi-square	*P*
**Terrestrial** ***Phytophthora*** **spp. (Non-clade 6 spp.)**														
Total annual precipitation	27.06	**<0.0001 (+)**	16.05	**0.00012 (+)**	8.48	**0.0036 (+)**	0.16	0.69 (**–**)	8.69	**0.0032 (+)**	3.8	0.051 (+)	4.6	**0.032 (+)**
Mean annual temperature	3.95	**0.047 (–)**	0.14	0.71 (**–**)	0.14	0.70 (+)	4.99	**0.025 (+)**	0.82	0.37 (**−**)	0.20	0.66 (+)	1.7	0.19 (+)
Land use	0.054	0.97	0.06	0.94	0.014	0.99	0.022	0.99	1.79	0.41	3.76	0.15	3.22	0.20
Year	24.39	**<0.0001 (+)**	16.4	**<0.0001 (+)**	11.31	**0.00077 (+)**	1.64	0.20 (+)	6.21	**0.013 (+)**	0.23	0.63 **(+)**	0.40	0.53 **(+)**
**Aquatic** ***Phytophthora*** **spp. (Clade 6 spp.)**														
Total annual precipitation	2.01	0.16 (−)	0.14	0.71 (−)	0.33	0.56 (**−**)	0.011	0.92 (+)	0.12	0.72 (+)	0.29	0.59 (−)	0.80	0.37 (**−**)
Mean annual temperature	0.15	0.69 (+)	4.57	**0.034 (−)**	4.56	**0.033 (−)**	7.71	**0.0055 (−)**	8.48	**0.0036 (−)**	6.4	**0.011 (−)**	7.51	**0.0061 (−)**
Land use	6.44	**0.04**	4.04	**0.020**	7.07	**0.029**	4.36	0.11	6.16	**0.046**	3.35	0.19	2.17	0.33
Year	16.4	**<0.0001 (+)**	29.78	**<0.0001 (+)**	27.98	**<0.0001 (+)**	15.49	**<0.0001 (+)**	5	**0.025 (+)**	31.57	**<0.0001 (+)**	13.24	**0.00027 (+)**

Significant *P*-values are shown in bold. “(+)” or “(−)” signs represent a positive or negative association with climatic factors, respectively. For the case of the factor “Year”, a positive sign indicates that the values were higher in the survey in 2014 than in 2013. For diversity parameters (species richness, Shannon index, Simpson index, and Pielou’s evenness), all operational taxonomic units (OTUs) identified at the species and clade level were included (total OTUs = 50). For functional diversity parameters (functional richness, functional dispersion, and functional evenness), only OTUs identified at the species level were included in the analysis (total OTUs = 36)

### Distribution of functional traits at the community and species level for terrestrial *Phytophthora* species

**Fig. 3:**
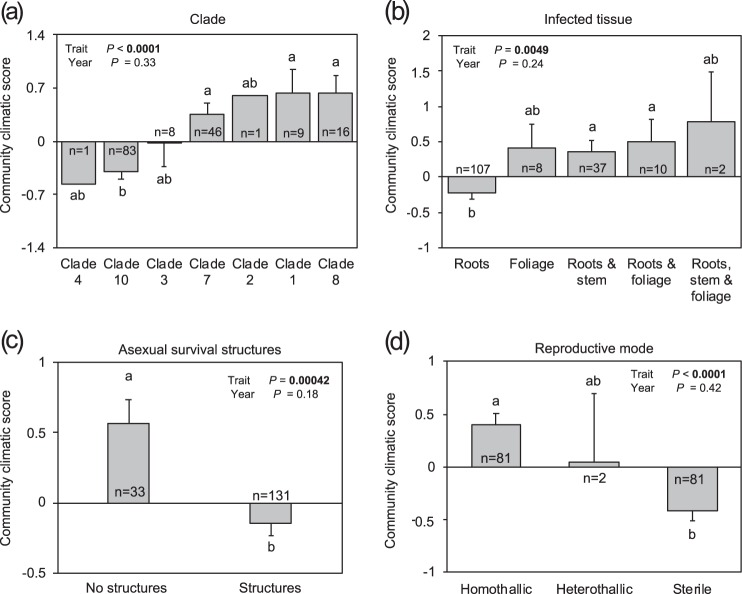
Distribution of terrestrial communities dominated by different trait values of **a** clade, **b** infected tissue, **c** asexual survival structures, and **d** reproductive mode, along a climatic gradient. Of the nine traits investigated, only the four traits with the highest *R*^2^ values are shown. Trait dominance was determined using the community-weighted mean (CWM) values of traits. A community climatic score with a value of 0 corresponds to the average temperature and precipitation values of all the sampling sites. In total, 164 terrestrial communities were included in the analysis. Error bars represent SE. Different lowercase letters above or below the error bars indicate significant differences at *P* < 0.05 when subjected to a protected Fisher least square differences (LSD) test. Higher values of community climatic score indicates wetter and warmer places, as showed in Figure 1

**Fig. 4:**
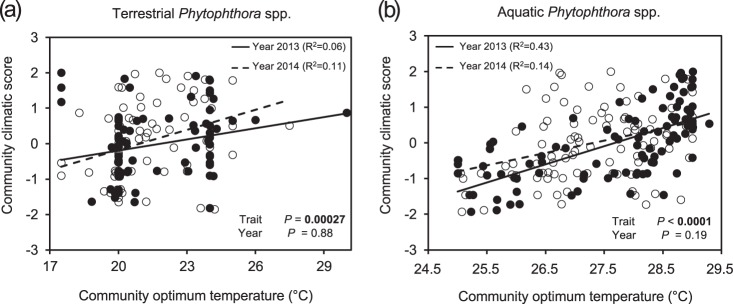
Community optimum temperature of **a** terrestrial and **b** aquatic *Phytophthora* communities along the climatic gradient. The community optimum temperature corresponds to the weighted mean of the optimum temperature for the species that comprise the community. A community climatic score with a value of 0 corresponds to the average temperature and precipitation values of all the sampling sites. Each circle corresponds to a community. Solid circles represent communities obtained in 2013; empty circles represent communities obtained in 2014. In total, 164 terrestrial and 192 aquatic communities were included in the analysis

We studied the association between climate and the dominance of functional traits of terrestrial *Phytophthora* species at the community level. All traits, except for the persistence of the sporangia, were associated with the climatic score. Communities in which the majority of the species belonged to phylogenetic clades 1, 7, or 8 were located in warmer and rainier areas than communities dominated by clade 10 species (Fig. 3a). Communities in which the most dominant *Phytophthora* species were only able to infect root tissue, were able to form asexual survival structures, or were sterile, were located in colder and drier areas than communities dominated by species with some other levels of the traits “infected tissue”, “asexual structures”, and “reproductive mode” (Fig. 3b–d). The weighted optimum temperature of the inland communities declined significantly with decreasing temperature and precipitation (Fig. 4a). Communities dominated by species with a wide host range were located in warmer and rainier areas than communities dominated by host-specific *Phytophthora* species (a mean climatic score of 0.46 versus −0.11 for wide host range and specific *Phytophthora* species, respectively; *P* = 0.0043).

We also studied whether temperature or precipitation accounted for more of the variation in the distribution pattern of each of the functional traits in the terrestrial communities. Temperature only accounted for more variation than precipitation in the case of species belonging to phylogenetic clades 2 or 8 (corresponding to the functional trait “clade”); for the rest of the traits analyzed, precipitation accounted for more variation than temperature (Supporting Information Table S7).

**Fig. 5:**
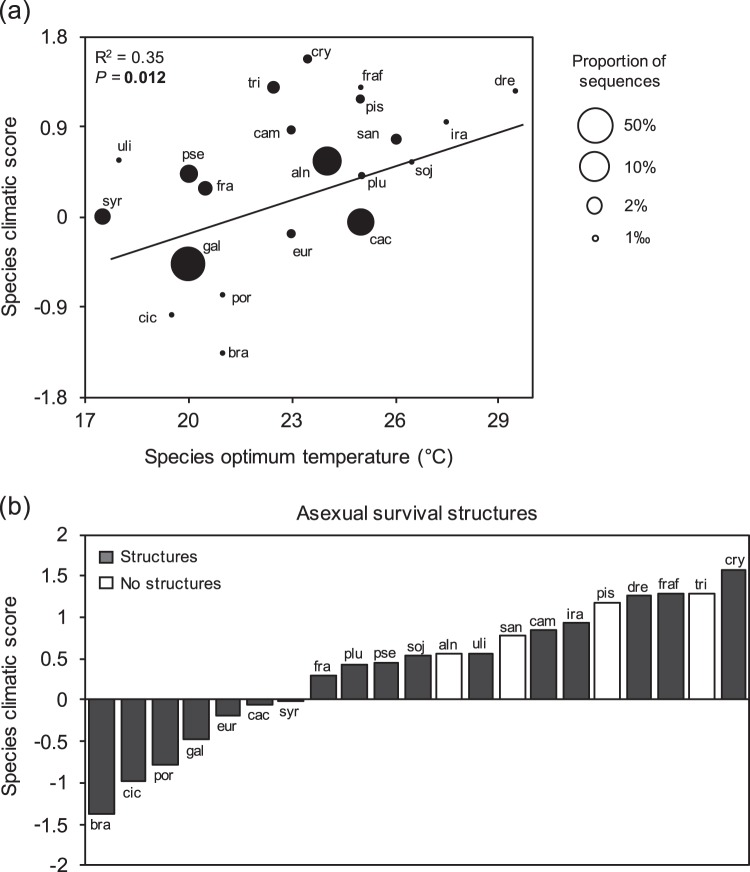
The association between climatic scores and functional traits at the species level for terrestrial *Phytophthora* species. **a** Association between the optimum temperature for the species and the species climatic score. The *R*^2^ and *P*-value (*P*) is based on a linear regression weighted by the proportion of total reads for each of the species. **b** Association between the climatic score and the asexual survival structures of terrestrial *Phytophthora* species detected at more than one location during the survey. The mean climatic score of species that form asexual structures was significantly higher than that of species that did not form structures (*P* = 0.02, Welch *t*-test). A climatic score with a value of 0 corresponds to the average temperature and precipitation values of all the sampling sites in the survey. In total, 21 *Phytophthora* species were included in this analysis: bra *P. brassicae*, cic *P. cichorii*, por *P. porri*, gal *P. gallica*, eur *P. europaea*, syr *P. syringae*, fra *P. fragariae,* plu *P. plurivora*, pse *P. pseudosyringae*, soj *P. sojae*, aln *P. alni* (species complex), uli *P. uliginosa*, san *P. sansomeana*, cam *P. cambivora*, ira *P. iranica,* pis *P. pisi*, dre *P. drechsleri*, fraf *P. fragariaefolia*, tri *P. trifolii*, cry *P. cryptogea*

At the species level, there was a significant association between the optimum temperature of each *Phytophthora* species and the species-climatic score (Fig. 5a). The average species-climatic scores of terrestrial *Phytophthora* species unable to form asexual survival structures were higher than those of species that were able to form asexual structures (0.94 versus 0.25, respectively; *P* = 0.02, Welch *t*-test) (Fig. 5b).

### Diversity and functional diversity of aquatic communities

Aquatic communities showed different diversity patterns than terrestrial communities. For instance, we found an increase in the level of alpha diversity (Shannon and Simpson index) with decreasing temperature and precipitation (Supporting Information Table S5), contrarily to what was found for terrestrial species. Furthermore, the land use of the site and the temperature accounted for more variation than precipitation for most of the diversity parameters (Table 2), while land use was not significant for terrestrial species. Sites located in urban or agricultural areas had significantly higher levels of species richness than forests, and sites located in agricultural areas had higher Shannon and Simpson index values than sites located in forests. The water chemistry was associated with the diversity of aquatic communities, and this association was not significant for terrestrial species. Total nitrogen and pH were positively correlated with the number of species and the Shannon index, respectively. Conductivity was negatively correlated with alpha diversity parameters (Shannon, Simpson, and evenness) (Supporting Information Table S6). The number of species and the alpha diversity of the samples collected in 2014 were higher than that of the samples collected in 2013 (Table 2).

The functional diversity of aquatic communities increased with decreasing temperature and precipitation, in contrast to terrestrial species (Supporting Information Table S5). Mirroring the patterns observed in terms of alpha diversity, temperature accounted for more functional diversity variation than the land use and precipitation (Table 2). Functional richness, dispersion and evenness increased significantly with decreasing temperature. Also, a water chemistry parameter showed a significant correlation with functional diversity in aquatic species, as water conductivity was negatively associated with functional dispersion (Supporting Information Table S6). Even though aquatic and terrestrial species were studied in the same sites, signs of environmental filtering were only found in 2.8% of the aquatic communities (Fig. 2b), which represented a 7 times lower value than what was observed for terrestrial species.

We studied the association between climate, water chemistry, and the dominance of functional traits of aquatic communities. Contrarily to terrestrial species, communities dominated by sterile species, species able to form asexual survival structures, or with a preference for root infections were located in warmer areas than communities dominated by some other levels of functional traits (Fig. S2). However, similarly to terrestrial *Phytophthora* species, the weighted optimum temperature of the community was positively correlated with the climatic score of the site (Fig. 4b). Nevertheless, and in contrast to terrestrial communities, the association between traits and climatic score that we observed at community level was not observed at the species level, since the association between certain functional traits and the species-climatic score was not significant.

## Discussion

### Diversity and functional diversity along a climatic gradient

Theory predicts that the diversity and functional diversity of living organisms decreases with the distance from the equator [11]. For soil fungi, several studies have shown that climatic factors explain the latitudinal patterns of diversity globally [13, 14], and the altitudinal patterns of diversity regionally [15]. In accordance with the literature, we showed for the first time that climatic factors are associated with the diversity and the functional trait distribution of microbial plant pathogens. Interestingly, we found that patterns of diversity along the climatic gradient were influenced by the physical environment where the pathogens completed their life cycle: water or soil. While diversity and functional diversity of terrestrial *Phytophthora* communities decreased with decreasing temperature and precipitation, the opposite was found for aquatic communities, showing increasing levels of diversity with decreasing temperature and precipitation. There are inconsistencies between studies regarding the relative importance of each of the climatic factors (temperature and precipitation) and the diversity of soil microorganisms [13, 14]. Our findings highlight the importance of the physical environment in which pathogens complete their life cycle, and may provide a mechanistic explanation for contrasting distribution patterns of other plant-associated microbes. In accordance with the results of Tedersoo et al. [13] for soil fungi, we showed that the diversity patterns of terrestrial *Phytophthora* communities were mainly associated with precipitation. Unlike terrestrial species, precipitation could be less of a constraint for aquatic *Phytophthora* species. Thus, in aquatic habitats, where water should not be limiting, we observed that temperature and water chemistry appeared to be stronger environmental drivers of diversity than precipitation. The importance of precipitation for the distribution of terrestrial *Phytophthora* plant pathogens is also consistent with their phylogenetic placement in the Stramenopila group within eukaryotes, with phylogenetically close organisms such as brown algae or diatoms, completing their life cycle in water [48]. Most terrestrial *Phytophthora* species are plant pathogenic, and therefore their patterns of distribution might be associated with plant diversity. Nevertheless, the number of tree species of the riverbank vegetation was not significant when included as a factor together with climatic factors (results not shown), supporting a predominant role climate on the patterns of diversity of *Phytophthora* communities in the studied area. However, different patterns than the ones observed in Sweden could be observed closer to the equator, and thus latitude may still be an aspect to consider when predicting different biotic or abiotic factors driving microbial diversity. We observed that functional richness was lower than expected based on the number of species in *c*. 20% of the examined terrestrial communities, and in *c*. 3% of the aquatic communities, which suggests that environmental filtering may be acting in northern latitudes, particularly on terrestrial species. The proportion of communities affected by environmental filtering seems consistent with the results of Lamanna et al. [16], who observed that environmental filtering had a decreasing effect among plant communities with increasing latitude (70% versus 40% in tropical and temperate forests, respectively).

### Distribution of functional traits of terrestrial *Phytophthora* species

The decline of functional richness, functional dispersion, and functional evenness with decreasing temperature and precipitation pointed to a convergence of traits in communities located in cold and dry areas. These results suggest that drought and cold temperatures favor the dominance of species exhibiting certain traits. Several studies have shown an association between specific functional traits and the distribution of organisms, such as plants [27] or lichens [49]; however, to the best of our knowledge, this has been rarely shown for plant-associated fungi, and never for plant pathogenic microorganisms. Within the genus *Phytophthora*, traits that converged in areas with harsh climatic conditions, such as the optimum temperature or the ability to create asexual survival structures, seemed to be related to the climatic suitability. Communities dominated by species with asexual survival structures such as chlamydospores or hyphal swellings were located in colder and drier regions than communities dominated by species that did not produce structures. These structures have been associated with the ability of *Phytophthora* species to tolerate low temperatures and/or drought periods [24, 25], thus structures allowing species to overcome adverse climatic conditions might be a pre-requisite for the establishment in northern latitudes. In a previous study, we found that the ability to create asexual structures was an important trait for the establishment of introduced *Phytophthora* species from nurseries into natural environments in Sweden [9]. Studies of mycorrhizal communities in boreal forests have shown that the most abundant root-associated ascomycetes in boreal forests have thick melanized cells allowing them to survive adverse environmental conditions [50, 51], indicating that surviving structures would be an important trait in northern latitudes irrespective of the trophic interaction established with the plant. Precipitation explained more variation than temperature with regard to the distribution of species able to create asexual structures, suggesting that asexual structures might play a stronger role in the tolerance to dry conditions than to cold. Sexual resistant structures have been associated with tolerance to cold for certain species such as *P.* x *alni* [10]. However, we found little support for this being a consistent pattern across the genus *Phytophthora* given that sterile species tended to dominate in colder and drier areas more than self-fertilizing homothallic species.

When studying highly uneven communities, community effects may be mistaken for the effects of the most dominant species in the community. To control for this putative caveat, we studied the association between the functional trait values and the distribution along the climatic gradient for each of the terrestrial species separately. We observed that the optimum temperature for growth and the ability to create asexual survival structures for individual terrestrial *Phytophthora* species were significantly associated with their distribution, regardless of their dominance in the community. These results open the door to the possibility of making predictions regarding the establishment and distribution of individual species based on traits, as has already been done for plants [29, 52, 53]. The association between the distribution of traits in terrestrial *Phytophthora* communities and climatic factors suggest that climate change could have an impact on the community assembly of these microorganisms. For instance, if the climate became warmer and wetter, communities currently dominated by specific root pathogens could be replaced by communities dominated by species with a wide host range that are able to colonize aerial tissues. Shifts in terms of incidence and distribution of certain *Phytophthora* species have been predicted based on climatic models [54–56]. Our results may provide relevant data to support future modeling and prediction of the distribution of *Phytophthora* species under climatic change conditions.

The most abundant terrestrial species reported in this study was *P. gallica*, a species that was previously isolated from water streams in surveys across Sweden [9]. The ecological role of this species remains unknown given the low number of reports of this species [57]. *P. gallica* was regarded as a specific pathogen of *Quercus robur* [45], although pathogenicity tests and surveys across North America showed its ability to colonize other hosts such as *Fagus sylvatica* and *Alnus* spp. [36, 57, 58]. The climatic score of *P. gallica* reported in this study was −0.47, indicating that its abundance was higher in areas of Sweden with temperature and precipitation levels below the average of the surveyed area. This finding, together with the previous reports of *P. gallica* in Alaska [58] may be an indication of the ability of this species to tolerate cold temperatures. By contrast, species displaying higher optimum temperatures than *P. gallica* such as *Phytophthora cryptogea*, *Phytophthora plurivora*, *Phytophthora drechsleri*, *Phytophthora iranica*, and *Phytophthora sojae*, had climatic scores higher than 0, indicating that their abundance was higher in areas of Sweden with temperatures above the average value of the surveyed area.

The abundance of particular species should be interpreted carefully. In the validation experiment, we found that species such as *P. gallica*, *Phytophthora pseudosyringae* or *P. gonapodyides* were underrepresented in the sequencing output of the mock community. Nevertheless, in the survey of the rivers, both baiting and sequencing showed very similar results. *P. gonapodyides* and *P. gallica* were amongst the five most abundant species isolated by baiting, and were also the second and fourth most abundant species after sequencing. Sampling both terrestrial and aquatic species in rivers might have affected our results, as terrestrial species may become diluted once in the river stream. Studies performing simultaneously baiting in streams and inland soils found that between 0 and 60% of the species recovered from soils, were also isolated from river water [36, 59, 60]. However, it seems that the dilution effect is much smaller by high-throughput sequencing than baiting. In the study of Catala et al., 90–100% of the species detected in soil were also detected in the nearby streams [35]. Sampling in streams can indeed be advantageous compared to direct soil sampling when studying communities along climatic gradients. Rivers seem to concentrate the inoculum of large areas, as suggested by the higher number of species detected in streams than in soil [35], possibly diluting microsite effects such as vegetation or soil type, that could affect soil communities. Even though some terrestrial species might have been missed during our survey, we found a great amount of terrestrial *Phytophthora* species (27 OTUs identified at species level), which belonged to all clades of *Phytophthora* except from clade 5 (a small clade containing only four species) [61]. Therefore, the patterns of diversity that we observed in this study are likely a good reflection a general pattern of terrestrial *Phytophthora* species at the genus level.

### Contrasting patterns of diversity and functional diversity between terrestrial and aquatic *Phytophthora* communities along a climatic gradient

We found that the diversity patterns of aquatic species differed from those of terrestrial communities. Given that water and soil are affected by temperature and precipitation in different ways, climate might affect microorganisms inhabiting these two environments in different ways. Indeed, we observed a lower frequency of environmental filtering among aquatic communities compared with that of terrestrial communities (2.8% versus 19.7% for aquatic and terrestrial communities, respectively), perhaps indicating a stronger buffering capacity of water versus soil against climatic adversities. Although we did not measure the temperature of the water, we assumed a positive correlation between air temperature and water temperature, as reported in other studies [62, 63]. Marine aquatic microorganisms, such as bacteria [64] or gastropods [65], have been reported to follow the same latitudinal diversity gradient patterns as terrestrial organisms; temperature appears to be the main driver of diversity, as we also observed in aquatic *Phytophthora* communities. However, the species richness of these marine aquatic microorganisms was higher in warmer environments, which is in contrast with our findings for freshwater communities, where a higher diversity was found in colder locations. Our results indicate that in fresh water, factors other than temperature may also influence diversity patterns. In our study, and in contrast to our findings for terrestrial communities, we observed a strong association between the land use of the sampling sites, the water chemistry, and the diversity and functional diversity of aquatic communities. Sampling sites located in urban areas had higher pH than forest sites, and urban and agricultural areas had higher water conductivity values and total nitrogen levels than forest sites (Supporting Information Table S8), indicating that the land use of the site influenced the quantity and quality of nutrients in the water, and therefore the composition of aquatic communities more than the communities of terrestrial species. Aram and Rizzo [34] showed that clade 6 species, particularly *P. gonapodyides*, have the ability to colonize dead plant tissue, suggesting that they may have a predominantly saprophytic lifestyle [30, 33, 34]. Therefore, it seems plausible that *Phytophthora* species of clade 6 would be more sensitive to nutrient availability and water chemistry than terrestrial species.

In conclusion, our results indicate that the association between climatic factors and diversity patterns, and the effect of environmental filtering associated with the assembly of *Phytophthora* communities, differs depending on where different *Phytophthora* species complete their life cycle. Our findings support previous studies suggesting that diversity in terrestrial and aquatic ecosystems is likely driven by different factors [39]. Functional traits can be used to predict distribution shifts under climate change scenarios both at the community and species level.

## Electronic supplementary material

Supplementary material
