# Supplementary material for: Contrasting distribution patterns between aquatic and terrestrial Phytophthora species along a climatic gradient are linked to functional traits
Source: ISME J. 2018 Aug 2;12(12):2967–80. doi: 10.1038/s41396-018-0229-3 (PMC6246556; doi:10.1038/s41396-018-0229-3)
Supplement: Supplementary file 1 — Supplementary material [file 41396_2018_229_MOESM1_ESM.pdf]

**Supporting Information**

Article title: Contrasting distribution patterns between aquatic and terrestrial *Phytophthora* species along a climatic gradient are linked to functional traits

Authors: Redondo, M.A., Boberg, J., Stenlid, J. and Oliva, J.

The next supporting information may be found in the online version of this article:

**Fig. S1** Maximum-likelihood phylogenetic tree of the ITS region for the phylogenetic clades 1-6 , and 7-10.

**Fig. S2.** Distribution of aquatic communities dominated by the different trait values of (a) infected tissue, (b) asexual survival structures, and (c) reproductive mode along a climatic gradient.

**Table S1** Sequence of primers used in the study

**Table S2** Results of the mock community

**Table S3** Comparison of the species obtained by high-throughput sequencing (PacBio) of river filtrates and the species obtained by traditional river baiting

**Table S4** *Phytophthora* traits used for the functional diversity calculations

**Table S5** Association between climatic score, land use of the site (urban, agricultural or forest), year of survey, and the diversity and functional diversity of terrestrial and aquatic *Phytophthora* communities

**Table S6** Association between the diversity and functional diversity parameters of terrestrial and aquatic communities, and the chemical parameters of the river water for the communities sampled during 2014

**Table S7** Association between temperature, precipitation, and the proportion of the terrestrial community for each level of functional trait

**Table S8** Values of chemistry parameters of the river water (pH, Conductivity, Total organic carbon, and Total nitrogen) for each of the land use types

**Methods S1** Methodology for the mock community analysis for the validation of primers.

**Methods S2** Criteria for selecting the functional traits of *Phytophthora* species.

(a)

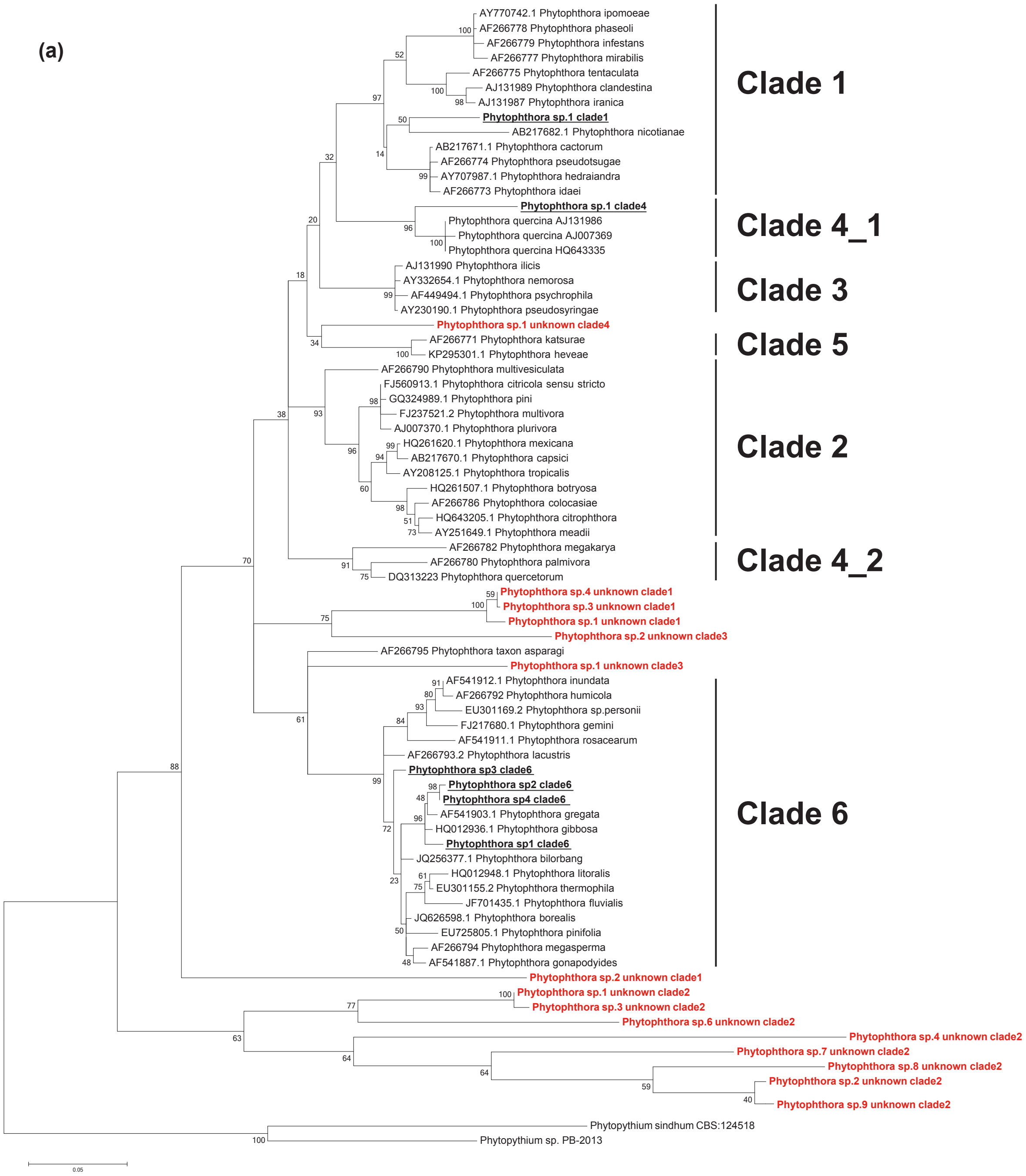
AY770742.1 Phytophthora ipomoeae
AF266778 Phytophthora phaseoli
AF266779 Phytophthora infestans
AF266777 Phytophthora mirabilis
AF266775 Phytophthora tentaculata
AJ131989 Phytophthora clandestina
AJ131987 Phytophthora iranica
Phytophthora sp.1 clade1
AB217682.1 Phytophthora nicotianae
AB217671.1 Phytophthora cactorum
AF266774 Phytophthora pseudotsugae
AY707987.1 Phytophthora hedraiandra
AF266773 Phytophthora idaei
Phytophthora sp.1 clade4
Phytophthora quercina AJ131986
Phytophthora quercina AJ007369
Phytophthora quercina HQ643335
AJ131990 Phytophthora ilicis
AY332654.1 Phytophthora nemorosa
AF449494.1 Phytophthora psychrophila
AY230190.1 Phytophthora pseudosyringae
Phytophthora sp.1 unknown clade4
AF266771 Phytophthora katsurae
KP295301.1 Phytophthora heveae
AF266790 Phytophthora multivesiculata
FJ560913.1 Phytophthora citricola sensu stricto
GQ324989.1 Phytophthora pini
FJ237521.2 Phytophthora multivora
AJ007370.1 Phytophthora plurivora
HQ261620.1 Phytophthora mexicana
AB217670.1 Phytophthora capsici
AY208125.1 Phytophthora tropicalis
HQ261507.1 Phytophthora botryosa
AF266786 Phytophthora colocasiae
HQ643205.1 Phytophthora citrophthora
AY251649.1 Phytophthora meadii
AF266782 Phytophthora megakarya
AF266780 Phytophthora palmivora
DQ313223 Phytophthora quercetorum
Phytophthora sp.4 unknown clade1
Phytophthora sp.3 unknown clade1
Phytophthora sp.1 unknown clade1
Phytophthora sp.2 unknown clade3
AF266795 Phytophthora taxon asparagi
Phytophthora sp.1 unknown clade3
AF541912.1 Phytophthora inundata
AF266792 Phytophthora humicola
EU301169.2 Phytophthora sp.personii
FJ217680.1 Phytophthora gemini
AF541911.1 Phytophthora rosacearum
AF266793.2 Phytophthora lacustris
Phytophthora sp3 clade6
Phytophthora sp2 clade6
Phytophthora sp4 clade6
AF541903.1 Phytophthora gregata
HQ012936.1 Phytophthora gibbosa
Phytophthora sp1 clade6
JQ256377.1 Phytophthora bilorbang
HQ012948.1 Phytophthora litoralis
EU301155.2 Phytophthora thermophila
JF701435.1 Phytophthora fluvialis
JQ626598.1 Phytophthora borealis
EU725805.1 Phytophthora pinifolia
AF266794 Phytophthora megasperma
AF541887.1 Phytophthora gonapodyides
Phytophthora sp.2 unknown clade1
Phytophthora sp.1 unknown clade2
Phytophthora sp.3 unknown clade2
Phytophthora sp.6 unknown clade2
Phytophthora sp.4 unknown clade2
Phytophthora sp.7 unknown clade2
Phytophthora sp.8 unknown clade2
Phytophthora sp.2 unknown clade2
Phytophthora sp.9 unknown clade2
Phytopythium sindhum CBS:124518
Phytopythium sp. PB-2013
Clade 1
Clade 4_1
Clade 3
Clade 5
Clade 2
Clade 4_2
Clade 6
0.05

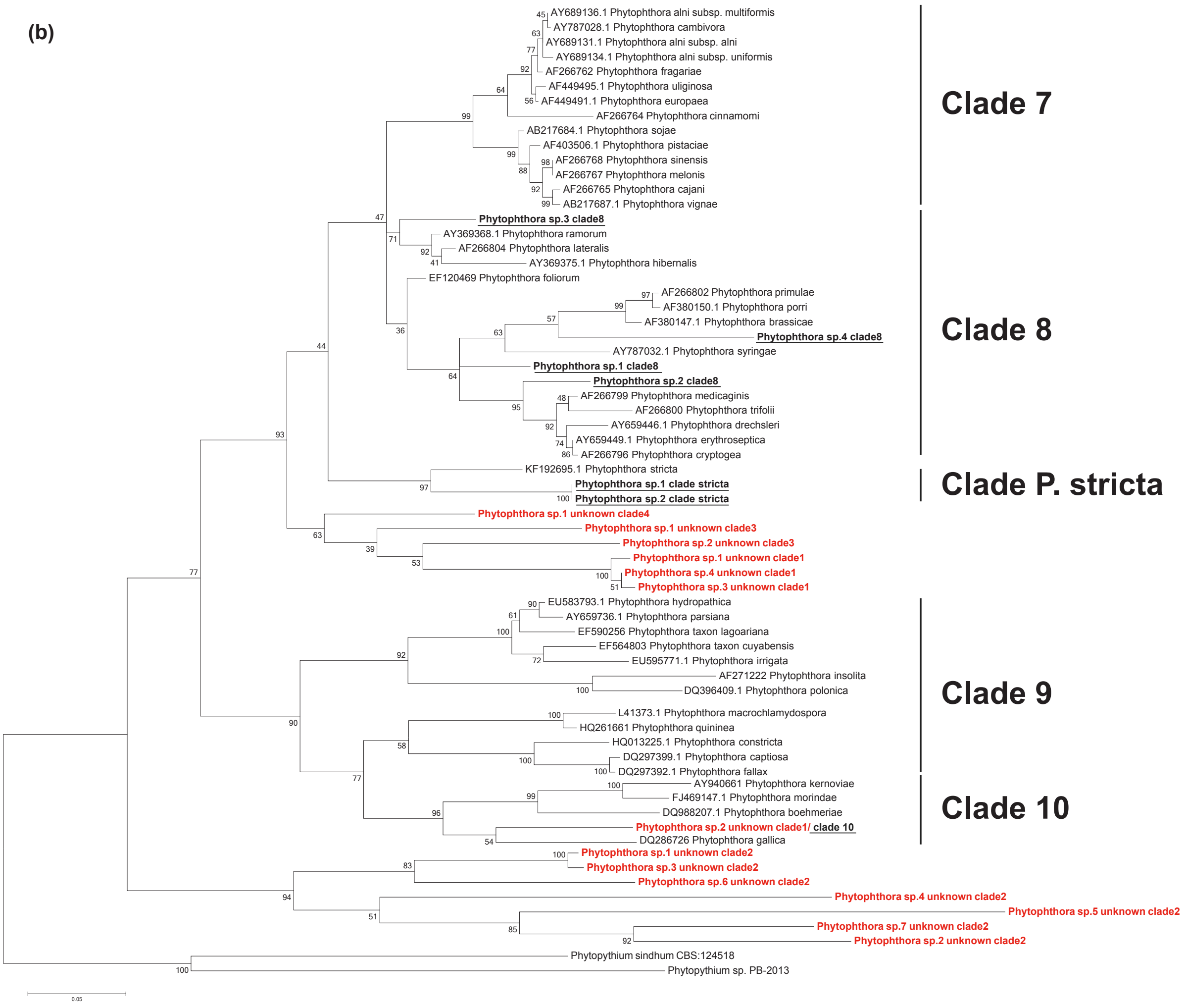


**Figure S1** Maximum-likelihood phylogenetic tree of the ITS region for the phylogenetic clades 1-6 (a), and 7-10 (b), including the sequences from Phytophthora ID database, and OTUs not identified at species level. Numbers before the branches represent the bootstrap scores. In boldface and, underlined, OTUs that were identified at clade level. In red, the OTUs that could not be assigned to any phylogenetic clade.

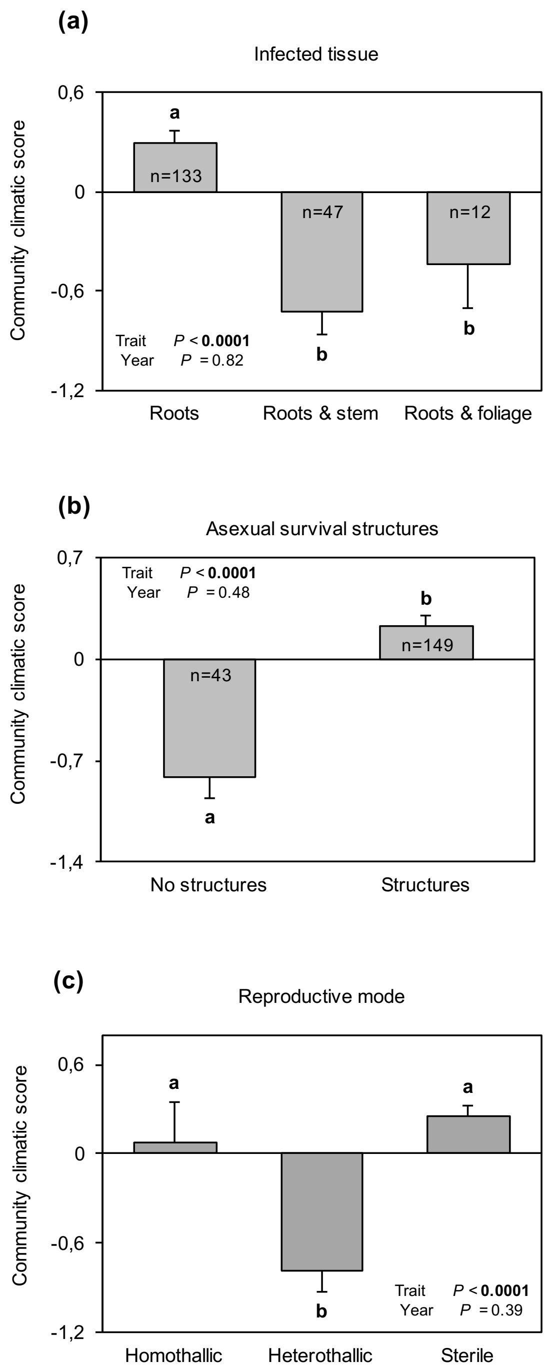


**Figure S2.** Distribution of aquatic communities dominated by different traits values of (a) infected tissue, (b) asexual survival structures, and (c) reproductive mode, along a climatic gradient. Only three out of 8 traits with higher $R^2$ value are shown. Trait dominance was obtained from Community Weighted Mean values of traits (CWM) from the FD package for R. Community climatic score with value = 0 corresponds to the average value of temperature

and precipitation of all the sampling sites. In total, 192 aquatic communities were included in the analysis. Error bars represent SE. Letters indicate significant differences at $P < 0.05$, with a protected Fisher least square differences (LSD) test.

**Table S1** Sequence of the primers used in this study.

| Primer name | Oligonucleotide sequence (5´-3´) | Specifications |
|---|---|---|
| I2-reverse | GATATCAGGTCCAATTGAGATGC | General for all *Phytophthora* spp. (Drenth *et al.* 2006) |
| A2-forward | ACTTTCCACGTGAACCGTTTCAA | General for all *Phytophthora* spp. (Drenth *et al.* 2006) |
| A2P-forward | ACTTTCCACGTGAACTGTATCGA | Primer modified to correct for mismatches with *P. pseudosyringae* |
| A2G-forward | ACTTTCCACGTGAACCGTATTAC | Primer modified to correct for mismatches with *P. gallica* |

**Table S2** Results of the mock community. *Phytophthora* species that were included in the mock community are displayed boldfaced.

| *Phytophthora* species | Number of reads | % of total reads | % identity with *Phytophthora* ID reference sequences |
|---|---|---|---|
| ***P.bilorbang*** | 1260 | 13.79% | 100 |
| ***P.plurivora*** | 1052 | 11.51% | 100 |
| ***P.ramorum*** | 964 | 10.55% | 100 |
| ***P.citrophthora*** | 904 | 9.89% | 100 |
| ***P.megasperma*** | 882 | 9.65% | 100 |
| ***P.syringae*** | 829 | 9.07% | 100 |
| ***P.lacustris*** | 812 | 8.88% | 99 |
| ***P.gregata*** | 715 | 7.82% | 99 |
| ***P.cactorum*** | 643 | 7.04% | 100 |
| ***P.gonapodyides*** | 568 | 6.22% | 99 |
| ***P.pseudosyringae*** | 422 | 4.62% | 100 |
| ***P.gallica*** | 82 | 0.90% | 100 |
| *P. riparia* | 4 | 0.04% | 99 |
| *P. uniformis* | 2 | 0.02% | 99 |

**Table S3** Comparison of the species obtained by high throughput sequencing (PacBio) of river filtrates and the species obtained by traditional river baiting. An asterisk "*" indicate aquatic *Phytophthora* species, i.e. species belonging to the phylogenetic clade 6.

| *Phytophthora* species | % of total reads (PacBio) | % of total isolates (baiting) |
|---|---|---|
| *P.alni* | 1.9% | - |
| *P.bilorbang** | 12% | 0.2% |
| *P.brassicae* | 0.02% | - |
| *P.cactorum* | 1.3% | 0.3% |
| *P.cambivora* | 0.04% | 0.8% |
| *P.capensis* | 0.007% | - |
| *P.cichorii* | 0.006% | - |
| *P.cryptogea* | 0.07% | 0.1% |
| *P.drechsleri* | 0.02% | - |
| *P.europaea* | 0.5% | - |
| *P.uliginosa* | 0.02% | - |
| *P.fragariae* | 0.3% | - |
| *P.fragariaefolia* | 0.01% | - |
| *P.gallica* | 6.5% | 1.0% |
| *P.gonapodyides** | 24.4% | 19.7% |
| *P.gregata** | 0.7% | 1.4% |
| *P.iranica* | 0.03% | - |
| *P.lacustris** | 50.4% | 72.6% |
| *P.sansomeana* | 0.1% | - |
| *P.megasperma** | 0.07% | 0.2% |
| *P.niederhauserii* | 0.004% | - |
| *P.chlamydospora** | 0.01% | - |
| *P.pinifolia** | 0.06% | - |
| *P.pisi* | 0.06% | - |
| *P.plurivora* | 0.01% | 3.3% |
| *P.porri* | 0.009% | - |
| *P.primulae* | 0.002% | - |
| *P.pseudosyringae* | 0.5% | - |
| *P.psychrophila* | 0.006% | - |
| *P.quercina* | 0.04% | - |
| *P.ramorum* | 0.01% | - |
| *P.riparia** | 0.2% | 0.3% |
| *P.rosacearum** | 0.1% | - |
| *P.sojae* | 0.02% | - |
| *P.syringae* | 0.4% | 0.1% |
| *P.trifolii* | 0.1% | - |

**Table S4** *Phytophthora* traits used for functional diversity calculations.

| Category | Trait | Type | Trait levels | Source |
|---|---|---|---|---|
| Life history | Clade | Factor | (A) Clade 1; (B) Clade 2; (C) Clade 3; (D) Clade 4; (E) Clade 5; (F) Clade 6; (G) Clade 7; (H) Clade 8; (I) Clade 9; (J) Clade 10 | Kroon *et al.* 2011 |
| | Reproductive mode | Factor | (A) Homothallic; (B) Heterothallic; (C) Sterile | Kroon *et al.* 2011 |
| | Persistence of sporangia | Factor | (A) Persistent; (B) Caducous; (C) Persistent and caducous | Kroon *et al.* 2011 |
| | Asexual survival structures | Factor | (A) No structures; (B) Chlamydospores; (C) Hyphal swellings; (D) Chlamydospores and hyphal swellings. | Erwin & Ribeiro 1996, Species description papers and expert knowledge |
| Environmental tolerance | Minimum temperature | Numeric | | Erwin & Ribeiro 1996, Species description, papers and expert knowledge |
| | Optimum temperature | Numeric | | Species description, papers |
| | Maximum temperature | Numeric | | Species description, papers |
| Specialization | Host range | Factor | (A) Genus specific; (B) Wide host range; (C) unknown | Kroon *et al.* 2011, and expert knowledge |
| | Infected tissue | Factor | (A) Roots; (B) Foliage; (C) Roots and stem; (D) Roots and foliage; (E) Roots, stem, and foliage; (F) Fruits; (G) Dead material (saprophytic *Phytophthora* spp.). | Kroon *et al.* 2011, and expert knowledge |

**Table S5** Association between climatic score (obtained from the PCA of temperature and precipitation), land use of the site (urban, agricultural or forest), year of survey, and diversity and functional diversity of terrestrial and aquatic *Phytophthora* communities. Significant P-values are displayed in boldface. “+” or “-“ signs in brackets represent a positive or negative association with climatic score. For the case of the factor “Year” a positive sign indicates that the diversity was higher in the year 2014 than in the year 2013. For diversity parameters (Species richness, Shanon index, Simpson index, Pielou´s evenness), all OTUs identified at species and clade level were included (total OTUs = 50). For functional diversity parameters (Functional richness, functional dispersion, and functional evenness), only OTUs identified at species level were included in the analysis (total OTUs = 36).

| | Species richness | | Shannon index | | Simpson index | | Pielou´s evenness | | Functional richness | | Functional dispersion | | Functional evenness | |
|---|---|---|---|---|---|---|---|---|---|---|---|---|---|---|
| | Chi-square | *P* | F value | *P* | Chi-square | *P* | Chi-square | *P* | Chi-square | *P* | Chi-square | *P* | Chi-square | *P* |
| Terrestrial *Phytophthora* spp. | | | | | | | | | | | | | | |
| Climatic score | 10.23 | **0.0014 (+)** | 11.32 | **0.0011 (+)** | 9.07 | **0.0026 (+)** | 2.06 | 0.15 (+) | 4.61 | **0.032 (+)** | 5.74 | **0.017 (+)** | 12.07 | **0.00051 (+)** |
| Land use | 0.24 | 0.88 | 0.0045 | 0.99 | 0.065 | 0.97 | 0.069 | 0.96 | 0.56 | 0.76 | 3.45 | 0.18 | 3.38 | 0.18 |
| Year | 22.88 | **< 0.0001 (+)** | 16.69 | **< 0.0001 (+)** | 11.69 | **0.00063 (+)** | 1.47 | 0.23 (+) | 8.49 | **0.0036 (+)** | 0.48 | 0.49 (+) | 0.32 | 0.57 (+) |
| Aquatic *Phytophthora* spp. | | | | | | | | | | | | | | |
| Climatic score | 1.12 | 0.29 (-) | 4.65 | **0.032 (-)** | 5.54 | **0.019 (-)** | 4.88 | **0.027 (-)** | 4.21 | **0.040 (-)** | 6.25 | **0.012 (-)** | 8.75 | **0.0031 (-)** |
| Land use | 7.92 | **0.019** | 3.66 | **0.028** | 6.46 | **0.039** | 4.35 | 0.11 | 4.34 | 0.11 | 3.08 | 0.22 | 1.23 | 0.54 |
| Year | 16.46 | **< 0.0001 (+)** | 29.63 | **< 0.0001 (+)** | 27.93 | **< 0.0001 (+)** | 15.25 | **< 0.0001 (+)** | 4.81 | **0.028 (+)** | 32.31 | **< 0.0001 (+)** | 13.71 | **0.0002 (+)** |

**Table S6** Association between diversity and functional diversity parameters of terrestrial and aquatic communities, and chemical parameters of the river water for the communities obtained during the year 2014. Significant P-values are displayed in boldface. “+” or “-“ signs in brackets represent a positive or negative association with water chemistry parameters. For diversity parameters (Species richness, Shanon index, Simpson index, Pielou´s evenness), all OTUs identified at species and clade level were included (total OTUs = 50). For functional diversity parameters (Functional richness, functional dispersion, and functional evenness), only OTUs identified at species level were included in the analysis (total OTUs = 36).

| | Species richness | | Shannon index | | Simpson index | | Pielou´s evenness | | Functional richness | | Functional dispersion | | Functional evenness | |
|---|---|---|---|---|---|---|---|---|---|---|---|---|---|---|
| | Chi-square | P | F value | *P* | Chi-square | *P* | Chi-square | *P* | Chi-square | *P* | Chi-square | *P* | Chi-square | *P* |
| Aquatic *Phytophthora* spp. | | | | | | | | | | | | | | |
| pH | 1.23 | 0.27 (+) | 4.39 | **0.039 (+)** | 4.54 | **0.033 (+)** | 1.81 | 0.18 (+) | 1.63 | 0.20 (-) | 3.14 | 0.076 (+) | 1.70 | 0.19 (+) |
| Conductivity | 0.036 | 0.85 (-) | 5.76 | **0.019 (-)** | 7.1 | **0.0078 (-)** | 3.93 | **0.047 (-)** | 0.086 | 0.77 (+) | 9.26 | **0.0023 (-)** | 1.46 | 0.23 (-) |
| Total organic carbon (TOC) | 1.98 | 0.16 (+) | 3.35 | 0.071 (+) | 3.69 | 0.055 (+) | 1.32 | 0.25 (+) | 0.32 | 0.57 (+) | 2.83 | 0.093 (+) | 2.35 | 0.12 (+) |
| Total nitrogen | 4.22 | **0.04 (+)** | 3.32 | 0.072 (+) | 2.78 | 0.095 (+) | 0.54 | 0.46 (+) | 1.41 | 0.24 (+) | 2.98 | 0.084 (+) | 0.018 | 0.89 (+) |
| Terrestrial *Phytophthora* spp. | | | | | | | | | | | | | | |
| pH | 0.0029 | 0.96 (+) | 0.071 | 0.79 (-) | 0.36 | 0.55 (-) | 0.45 | 0.50 (-) | 0.95 | 0.33 (+) | 0.041 | 0.84 (-) | 1.44 | 0.23 (-) |
| Conductivity | 3.39 | 0.065 (-) | 0.89 | 0.35 (-) | 0.048 | 0.83 (-) | 0.55 | 0.46 (+) | 3.06 | 0.081 (-) | 0.011 | 0.92 (-) | 0.36 | 0.55 (+) |
| Total organic carbon (TOC) | 0.0074 | 0.93 (-) | 0.43 | 0.51 (-) | 0.92 | 0.34 (-) | 0.99 | 0.32 (-) | 0.63 | 0.43 (+) | 1.55 | 0.69 (-) | 1.31 | 0.25 (-) |
| Total nitrogen | 1.88 | 0.17 (+) | 0.64 | 0.43 (-) | 0.037 | 0.85 (+) | 0.40 | 0.53 (-) | 0.93 | 0.33 (+) | 0.01 | 0.92 (-) | 0.33 | 0.56 (-) |

**Table S7** Association between temperature, precipitation, and the proportion of the terrestrial community with each level of functional trait. For functional traits that were categorical or binary, each trait level was converted to a dummy variable: proportion of community of that specific level of trait. Significant P-values are displayed in boldface. "+" or "-" signs in brackets represent a positive or negative association with climatic factors.

| | | Mean annual temperature | | | Total annual precipitation | |
|---|---|---|---|---|---|---|
| | Chi-square | F-value | P | Chi-square | F-value | P |
| Clade | | | | | | |
| Clade 1 | 0.042 | - | 0.84 (-) | 4.67 | - | **0.031 (+)** |
| Clade 2 | 6.82 | - | **0.009 (+)** | 0.63 | - | 0.43 (-) |
| Clade 3 | 6.01 | - | **0.014 (-)** | 9.82 | - | **0.0017 (+)** |
| Clade 4 | 1.90 | - | 0.17 (+) | 7.04 | - | **0.008 (-)** |
| Clade 7 | 0.18 | - | 0.67 (-) | 11.60 | - | **0.00066 (+)** |
| Clade 8 | 4.28 | - | **0.039 (+)** | 0.39 | - | 0.53 (-) |
| Clade 10 | 0.041 | - | 0.84 (-) | 29.36 | - | **< 0.0001 (-)** |
| Reproductive mode | | | | | | |
| Homothallic | 0.032 | - | 0.86 (-) | 29.18 | - | **<0.0001 (+)** |
| Heterothallic | 1.91 | - | 0.17 (+) | 0.11 | - | 0.75 (+) |
| Sterile | 0.041 | - | 0.84 (-) | 29.36 | - | **< 0.0001 (-)** |
| Asexual survival structures[1] | | | | | | |
| No structures | 1.60 | - | 0.21 (+) | 6.17 | - | **0.013 (+)** |
| Chlamydospores[1] | 0.1 | - | 0.75 (-) | 18.18 | - | **< 0.0001 (-)** |
| Hyphal swellings[2] | 1.04 | - | 0.31 (-) | 11.29 | - | **0.00078 (-)** |
| Minimum temperature | - | 2.25 | 0.14 (+) | - | 13.17 | **0.00038 (-)** |
| Optimum temperature | - | 2.05 | 0.15 (+) | - | 7.82 | **0.0058 (+)** |
| Maximum temperature | - | 2.21 | 0.14 (+) | - | 15.14 | **0.00015 (-)** |
| Wide host range | 0.87 | - | 0.35 (+) | 6.43 | - | **0.011 (+)** |
| Tissue[2] | | | | | | |
| Roots | 1.1 | - | 0.74 (-) | 0.015 | - | 0.9 (-) |
| Stem | 0.072 | - | 0.79 (-) | 8.78 | - | **0.003 (+)** |
| Foliage | 0.21 | - | 0.65 (+) | 4.35 | - | **0.037 (+)** |
| Fruits | 28.33 | - | **< 0.0001 (-)** | 66.93 | - | **< 0.0001 (-)** |

[1]Species that can create more than one type of structures were added in each of the models for the structures, i.e. species that can create both chlamydospores and hyphal swellings were added both in the chlamydospores and in the hyphal swellings model.

[2]Species that can infect more than one tissue were added in each of the models for the tissues that can infect. For instance, species that can infect roots and stem, were added both in the root and in the stem model.

**Table S8** Values of chemistry parameters of the river water (pH, Conductivity, Total organic carbon, and Total nitrogen) for each of the land use types. Letters indicate significant differences at *P* < 0.05, with a protected Fisher least square differences (LSD) test.

| | pH | | Conductivity | | Total organic carbon | | Total nitrogen[1] | |
|---|---|---|---|---|---|---|---|---|
| Land use | | | | | | | | |
| Urban | 7.2 | a | 20.2 | a | 13.5 | a | 943.9 | a |
| Agricultural | 7 | ab | 19.2 | a | 16.8 | a | 1012.3 | a |
| Forest | 6.8 | b | 10.6 | b | 17.7 | a | 742.5 | a |

[1]For the statistical analysis, total nitrogen was log transformed to fulfill the normality assumptions. In this analysis, the land use was marginally significant (P = 0.052).

**Methods S1** Mock community preparation for validation of primers

To test the validity of the primers used in this study, we prepare a mock community including 12 *Phytophthora* species (Table S2). DNA was extracted from single tip cultures with the NucleoSpin® Plant II kit (Macherey-Nagel, Hoerdt, France), and quantified with the Qubit fluoroometer. An equimolar pool of all 12 species was done and, aliquots of the pool were made at 0.001ng/ul, 0.01ng/ul and 0.1 ng/ul to obtain the optimum concentration, as explained in Material and Methods. Three replicates of the pool of species at the optimum concentration were amplified for 20 cycles, using the primers and PCR program described before. The PCR product of the three replicates was pooled and purified with the E.Z.N.A. Cycle Pure Kit (Omega bio-tek, Norcross, GA, USA). Adaptor ligation and Pacific Biosciences PSII sequencing was done at SciLifeLab (NGI, Uppsala) in 1 SMRT cell. We obtain a total of 9139 *Phytophthora* reads after removing tag jumps (i.e. artefact reads with different tags in the forward and reverse primers) and singletons.

**Methods S2** Criteria for selection of functional traits of *Phytophthora* species.

The functional traits of the *Phytophthora* species were obtained from the literature (Table S1). In order to deal with inconsistencies, the following criteria were used:

For asexual survival structures, we considered that a species can create structures if (i) they have been observed by the authors of the species description, even if it has been observed sporadically, or (ii) asexual survival structures where noted by three or more independent studies.

When minimum, optimum or maximum temperature of growth were reported as a range of temperatures, we took the mean value of the range. Also, we considered that the minimum and maximum temperatures refer to the temperature at which growth does not occur, which does not imply that the *Phytophthora* species is dead at that temperature.

Only field reports were considered as valid for tissue preference. Tissues infected in laboratory conditions or baiting were disregarded.

Reproductive mode was defined by behavior in V8 media. A species was considered homothallic when it was able to self-fertilize. Heterothallic when the colony only produced oospores in presence of a compatible mating type from the same or from another species. A species was defined as sterile if oospores were not produced in any of the previous conditions.
